# Prediction of Long-term Post-operative Testosterone Replacement Requirement Based on the Pre-operative Tumor Volume and Testosterone Level in Pituitary Macroadenoma

**DOI:** 10.1038/srep16194

**Published:** 2015-11-05

**Authors:** Cheng-Chi Lee, Chung-Ming Chen, Shih-Tseng Lee, Kuo-Chen Wei, Ping-Ching Pai, Cheng-Hong Toh, Chi-Cheng Chuang

**Affiliations:** 1 Institute of Biomedical Engineering, National Taiwan University, Taiwan, ROC; 2 Department of Neurosurgery, Chang Gung Memorial Hospital, Chang Gung University, Taoyuan, Taiwan, ROC; 3 Radiation Oncology, Chang Gung Memorial Hospital, Chang Gung University, Taoyuan, Taiwan, ROC; 4 Medical Imaging and Intervention, Chang Gung Memorial Hospital, Chang Gung University, Taoyuan, Taiwan, ROC

## Abstract

Non-functioning pituitary macroadenomas (NFPAs) are the most prevalent pituitary macroadenomas. One common symptom of NFPA is hypogonadism, which may require long-term hormone replacement. This study was designed to clarify the association between the pre-operative tumor volume, pre-operative testosterone level, intraoperative resection status and the need of long-term post-operative testosterone replacement. Between 2004 and 2012, 45 male patients with NFPAs were enrolled in this prospective study. All patients underwent transsphenoidal surgery. Hypogonadism was defined as total serum testosterone levels of <2.4 ng/mL. The tumor volume was calculated based on the pre- and post-operative magnetic resonance images. We prescribed testosterone to patients with defined hypogonadism or clinical symptoms of hypogonadism. Hormone replacement for longer than 1 year was considered as long-term therapy. The need for long-term post-operative testosterone replacement was significantly associated with larger pre-operative tumor volume (p = 0.0067), and lower pre-operative testosterone level (p = 0.0101). There was no significant difference between the gross total tumor resection and subtotal resection groups (p = 0.1059). The pre-operative tumor volume and testosterone level impact post-operative hypogonadism. By measuring the tumor volume and the testosterone level and by performing adequate tumor resection, surgeons will be able to predict post-operative hypogonadism and the need for long-term hormone replacement.

Non-functioning pituitary macroadenomas (NFPAs) are the most prevalent pituitary macroadenomas. Although NFPAs are benign in origin, mass effects may lead to significant clinical symptoms, such as visual impairments and pituitary insufficiency. It is also well known that pituitary function may be affected by this primary mass effect. The pre-operative tumor volume, especially in cases of larger tumors, may significantly impact pre-operative hypogonadism and post-operative recovery from this deficiency. Dekkers *et al.* have reported that, in patients presenting with hypopituitarism, growth hormone (GH) deficiency and gonadal deficiency are observed in approximately 85% and 75% of all patients, respectively; alternatively, corticotroph (approximately 38%) and thyrotroph (approximately 32%) deficiencies are observed less frequently^1^,^2^,^3^,^4^,^5^,^6^,^7^,^8^. Moreover, Losa *et al.* have reported that diabetes insipidus occurred in 5.9% of their patients, and true cases of permanent diabetes insipidus requiring life-long replacement therapy with desmopressin composed 1.6% of their sample^9^. However, because GH deficiency typically does not have a significant clinical impact, supplements for GH insufficiency are not prescribed unless the patient presents with a severe, clinically manifesting GH deficiency^10^,^11^,^12^. Gonadal function is the most sensitive endocrine axis of the pituitary gland; thus, it is the most vulnerable to external mass compression. Patients with hypogonadism may present with decreased libido, bone density, muscle mass/strength, and fatigue that interfere with their daily activities^13^. Therefore, our study focused on hypogonadism and subsequent testosterone supplementation in male patients. Although permanent impairment of pituitary endocrine function has been reported to be caused by tumors, apoplexy, or surgical manipulations, surgery remains the treatment of choice for NFPAs. Additionally, relieving the pressure on the normal pituitary may promote post-operative recovery from hypopituitarism. Arafah has reported that improvement in pituitary function occurred more often in patients with tumors measuring ≤25 mm in diameter than in patients with larger tumors^14^. However, Webb *et al.* have stated that, in both functioning pituitary macroadenomas and NFPAs, the tumor size did not differ significantly between patients who did and who did not recover pituitary function after surgery^15^. Regardless of size, a one-dimensional measurement (diameter alone) is not as accurate as a three-dimensional measurement (volume) in predicting post-operative pituitary function. Moreover, Webb *et al.* have also noted that endocrine recovery tended to be less common in patients with NFPAs after subtotal tumor resection (STR, 26% post-operative recovery) than after gross total tumor resection (GTR, 44% recovery), although this difference did not reach statistical significance (p = 0.146)^15^. Therefore, whether the pre-operative tumor volume or intraoperative resection ratio significantly impacts post-operative hormone deficiency and whether long-term supplementation is needed warrant further investigation. To clarify the impact of the tumor volume and resection ratio on hypogonadism, we sought to quantify this correlation by calculating the most accurate pre- and post-operative NFPA volume by using a more accurate method. We also calculated the resection/residual tumor ratio accordingly. The aim of this study was to assess the relationship between the pre-operative tumor volume, the resection/residual ratio, and hypogonadism and, if possible, to predict the necessity of long-term hormone replacement.

## Results

### Patient Subgroups

Between 2004 and 2012, 45 male patients with NFPAs underwent a total of 52 operations; 7 patients underwent a second operation because of residual tumor progression. As shown in Table 1, 29 cases (55.8%) were in the large tumor group (LTG), whereas 23 cases (44.2%) were in the small tumor group (STG). We performed STR in 24 cases (45.8%) and GTR in the remaining 28 cases (54.2%). Post-operative magnetic resonance imaging (MRI) revealed a residual tumor ratio of <0.22 in 40 cases (76.9%) and ≥0.22 in 12 cases (23.1%). The pre-operative testosterone level was <1.5 ng/mL in 28 cases (53.8%) and ≥1.5 ng/mL in the remaining 24 cases (46.2%).

### Correlation Coefficient between the Pre-operative Tumor Volume and the Serum Testosterone Level

**Figure 1:**
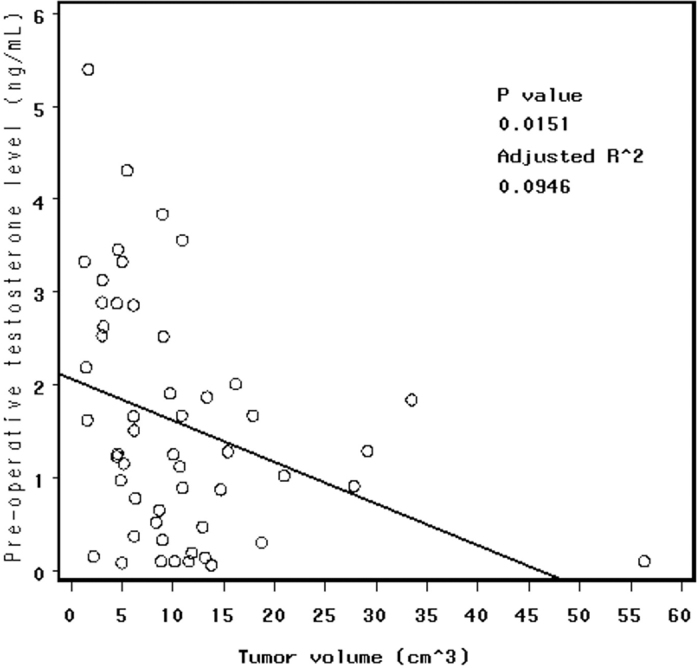
Scatter diagram showing that a larger pre-operative tumor volume negatively impacts the pre-operative serum level of testosterone, with a coefficient of −0.335 (p = 0.0151, adjusted R^2^ = 0.0946), based on the Pearson correlation coefficients method.

We found that a larger pre-operative tumor volume was negatively associated with the pre-operative serum testosterone level, as demonstrated by the scatter diagram shown in Fig. 1. We also calculated Pearson correlation coefficients, yielding a coefficient of −0.335 (p = 0.0151, adjusted R^2^ = 0.0946), and this result confirmed a negative relationship between the pre-operative tumor volume and the pre-operative testosterone level.

### Subgroups Receiving Long-term Testosterone Replacement

The details about patients in different subgroups receiving long-term testosterone replacement are shown in Table 2. Most cases in the LTG (18/29, 62.1%) required long-term testosterone supplementation. Of the 12 cases in which the intraoperative residual ratio was ≥0.22, the majority (8/12, 66.7%) required testosterone supplementation. Of the 28 cases in which the pre-operative testosterone level was <1.5 ng/mL, 19 cases (67.9%) required testosterone supplementation. Moreover, of all 52 cases, 22 (42.3%) required long-term testosterone therapy, and 48 of the 52 cases (92.3%) were diagnosed with GH deficiency. Furthermore, in all 22 cases that required long-term testosterone supplementation, 11 (50%) required concurrent long-term cortisone supplementation. Among these 11 cases, 2 (9.09%) required long-term thyroxine replacement, another case (4.55%) required desmopressin supplementation, and a third case (4.55%) required simultaneous thyroxine and desmopressin treatment.

### Univariate Logistic Regression and Categorical Analyses

To determine the cut-off value with the greatest sensitivity and most significant p value for predicting the need for long-term testosterone supplementation, we applied univariate logistic regression analysis. As shown in Table 3, when considering the tumor volume as a continuous variable, we found that the best sensitivity (0.8181) and an acceptable area under the receiver operating characteristic (ROC) curve (0.726) as well as the most significant p value (p = 0.0022) existed simultaneously at volumes of 7 and 8 cm^3^. As shown in Table 4, there was no obvious cut-off value of the residual ratio to predict the need for long-term testosterone replacement. As shown in Table 5, when considering the pre-operative testosterone level as a continuous variable, we found that at levels of 1.3, 1.4, and 1.5 ng/mL, the best sensitivity (0.8636), most acceptable area under the ROC curve (0.782), and most significant p value (p = 0.0003) existed simultaneously. As shown in Table 6, with regard to the categorical variables, also including the intraoperative resection status and patients who underwent second surgery or not, as our potential risk factors, chi-square and Fisher’s exact tests showed that there were significant differences in the need for long-term testosterone replacement based on the following cut-off values: tumor volume of 7 cm^3^ (p = 0.0012) and pre-operative testosterone level of 1.5 ng/mL (p < 0.0001). There were no statistically significant differences regarding the need for long-term testosterone replacement between the STR and GTR groups (p = 0.2986), in the residual ratio (cut-off value: 0.22, p = 0.0515) and in second surgery (p = 0.9999).

### Evaluation of the Relationship between the Pre-operative Tumor Volume, the Serum Testosterone Level, the Residual Tumor Ratio, the Resection status, Second Surgery, and the Need for Long-term Testosterone Therapy by Using Multivariate Logistic Regression Analysis

As shown in Table 7, when considering multiple variables together and the need for long-term testosterone therapy, we found that there were significant differences in the larger pre-operative tumor volume (p = 0.0067), the lower pre-operative testosterone level (p = 0.0101), and patients who underwent second surgery or not (p = 0.0250). There were no statistically significant differences in the resection status (p = 0.1059) or in the higher residual tumor ratio (p = 0.1040). As shown in Table 8, when considering the three significant factors shown in Table 7 together, we found that there were also significant differences in the larger pre-operative tumor volume (p = 0.0082), the lower pre-operative testosterone level (p = 0.0055), and patients who underwent second surgery or not (p = 0.0257). As shown in Table 9, we found that there were significant differences in the need for long-term testosterone therapy based on the following cut-off values: pre-operative tumor volume of 7 cm^3^ (p = 0.0144) and pre-operative testosterone level of 1.5 ng/mL (p = 0.0015). There was no statistically significant difference in patients who underwent second surgery or not (p = 0.1190). The choices and determination of the cut-off values will be discussed later.

### Analysis of the Association between Risk Factors Associated with Long-Term Testosterone Therapy by Using Multivariate Logistic Regression Stratified by the Pre-Operative Tumor Volume

With regard to the residual tumor ratio, although it was determined to be an insignificant risk factor by the previous statistical results, the stratification analysis as shown in Table 10 revealed diverse statistically significant differences between the stratified pre-operative tumor volumes of <7 cm^3^ (p = 0.2723, insignificant) and ≥7 cm^3^ (p = 0.0367, significant) when regarding the higher residual tumor ratio as a risk factor of the need for long-term testosterone therapy. The same result was found when the stratified volume was at 8 cm^3^. Furthermore, the results of statistical analyses were not so diverse between volumes <9 cm^3^ (p = 0.3267, insignificant) and ≥9 cm^3^ (p = 0.0429, slightly significant). Moreover, the statistical analyses did not yield significant differences or diverse results between tumors with volumes of <10 cm^3^ (p = 0.3335) and ≥10 cm^3^ (p = 0.0608) and between tumors with volumes of <11 cm^3^ (p = 0.3617) and ≥11 cm^3^ (p = 0.1855). With regard to second surgery, there were no statistically significant differences between any of the subgroups stratified by the pre-operative tumor volume.

## Discussion

Pituitary adenomas are benign tumors that arise from adenohypophyseal cells and represent 10–20% of all intracranial tumors^16^. NFPAs are often not diagnosed until they are sufficiently large to compress adjacent anatomical structures and cause visual disturbances, headaches, and impaired pituitary function^7^,^14^,^17^,^18^. In patients with macroadenomas, hypopituitarism can be caused by three mechanisms: 1) compression of the pituitary stalk, which causes decreased availability of hypothalamic stimulatory hormones; 2) compression of functioning pituitary tissue; and 3) hypothalamic involvement of the pituitary tumor^19^.

Transsphenoidal surgery achieves the goal of alleviating endocrine, visual, and other neurological defects by removing as much of the tumor as possible. The studies summarized in Table 11 included surgically treated pituitary macroadenomas, the majority of which were clinically non-functioning. After surgical decompression, recovery from hypopituitarism is possible if normal pituitary tissue is remaining^15^, and residual anterior pituitary function can be preserved and even improved after transsphenoidal surgery. If the pituitary tissue has been destroyed, the recovery of normal function is unlikely, and lifelong hormone replacement therapy is required. Similar results have been obtained in recent studies^20^,^21^,^22^.

### Pre-Operative Hypogonadism

In patients presenting with hypopituitarism, gonadal function was the most frequently observed impairment. Based on the mechanisms described above, the mass effect is clearly the dominating cause of pituitary insufficiency, and gonadal function is the most vulnerable axis to external mass compression. Therefore, we focused on hypogonadism. Statistical analyses revealed that a larger tumor volume negatively impacted pre-operative hypogonadism (Fig. 1), which typically indicates a lower pre-operative testosterone level (Pearson correlation coefficient: −0.335, p = 0.0151, adjusted R^2^ = 0.0946). Therefore, a larger tumor volume would tend to cause more severe pre-operative hypogonadism. Although the pre-operative testosterone level is also a significant risk factor for post-operative testosterone replacement (p = 0.0101), we speculate that the tumor volume may be the primary factor causing long-term post-operative testosterone deficiency and that pre-operative hypogonadism may be only a secondary effect of the tumor volume. However, the pre-operative testosterone level could still be used as a predictor of the need for post-operative hormone supplementation.

### Hypogonadism after Surgery

Dekkers *et al.* have reported that 90% of their patients were deficient in luteinizing hormone (LH) or follicle-stimulating hormone (FSH), 83% were GH-deficient, 60% were adrenocorticotropic hormone-deficient, and 57% were thyroid stimulating hormone (TSH)-deficient post-operatively^1^. Our study revealed that, of the 52 cases, 22 (42.3%) required long-term testosterone therapy and 92.3% were also diagnosed as GH-deficient; however, we did not prescribe supplementation for GH deficiency as described above. We found that, of the 22 cases requiring testosterone replacement, 11 (50%) required concurrent long-term cortisone supplementation. Among these 11 cases, 2 cases (9.09%) required long-term thyroxine replacement, another case (4.55%) required desmopressin supplementation, and a third case (4.55%) required simultaneous thyroxine and desmopressin treatment. In agreement with results from other studies, gonadal function was the most frequently affected hormonal axis that required replacement therapy both before and after surgery. Some studies have reported variable degrees of improvement in pituitary function after surgery^4^,^6^,^8^,^15^,^23^,^24^, whereas other studies have been unable to demonstrate a significant improvement in pituitary function^2^,^7^ or even have reported decreased pituitary function after transsphenoidal surgery^2^,^16^,^25^. Whether transsphenoidal surgery helps to improve pre-operative hypopituitarism remains a matter of debate. We propose two reasons for this uncertainty. First, although it is widely understood that larger tumors cause a lower pre-operative testosterone level, the exact cut-off value of the tumor volume for predicting recovery from hypogonadism is unknown. Therefore, even if adequate decompression is achieved in a patient whose tumor volume exceeds the cut-off value (e.g., 7 cm^3^ in our study), hypogonadism may not be ameliorated after the surgery. Second, excessively aggressive and blind surgical resection could result in unwanted pituitary gland damage.

### Prediction of Post-Operative Pituitary Function

Before and during the operation, the prediction of post-operative pituitary function is important to enable the evaluation of whether the pituitary tissue will remain functionally viable after adequate surgical resection.

#### Determination of the Cut-Off Value

As we mentioned above, when considering tumor volume as the risk factor, the univariate logistic regression analysis produced the same results at volumes of 7 and 8 cm^3^. Although the same statistical results occurred for both volumes, a tumor volume larger than 7 cm^3^ may be associated with a risk of long-term hypogonadism at a lower threshold at an earlier time than a tumor volume ≥8 cm^3^. Thus, 7 cm^3^ may represent the optimal cut-off value of the tumor volume associated with the recovery of gonadal function, and this threshold may predict the necessity of long-term hormone replacement. Conversely, when considering the pre-operative testosterone level as a risk factor, the univariate logistic regression analysis produced the same results at levels of 1.3, 1.4, and 1.5 ng/mL. Although the same statistical results were obtained in these three situations, a pre-operative testosterone level <1.5 ng/mL may predict a risk of long-term hypogonadism at a lower threshold at an earlier time than a level <1.3 ng/mL. Thus, 1.5 ng/mL may also represent the optimal cut-off value of the pre-operative testosterone level associated with the recovery of gonadal function, and this threshold may also predict the necessity of long-term testosterone therapy. Regarding the residual tumor ratio, although a ratio of 0.22 was associated with a p value of 0.0595 and an odds ratio (OR) of 3.714 (0.949–14.541), the residual ratio was not such a strong risk factor in predicting the need for testosterone supplementation based on this statistical result. However, we still recognize the residual ratio as one of the potential risk factors influencing the need for post-operative long-term testosterone supplementation, as will be discussed in the following section.

#### Pre-operative Tumor Size and Volume

Arafah has observed more frequent improvement in pituitary function in patients with tumors measuring 2.5 cm or less than in patients with larger tumors^14^. However, Webb *et al.* have stated that in both functioning pituitary macroadenomas and NFPAs, the tumor size was not significantly associated with the improvement of pituitary function^15^; however, these authors divided the tumors into microadenoma and macroadenoma groups using a cut-off tumor diameter of 1 cm. Therefore, some tumors sized between 1 and 2.5 cm, which were categorized as macroadenomas in their study, may not exert a significant mass effect on the gland and may not require hormone replacement. Regardless of the aforementioned findings, the one-dimensional method of tumor measurement is not scientifically convincing. Therefore, to more accurately and scientifically assess these factors, we adopted a more quantitative volumetric method to calculate the tumor volume and determined the potential cut-off value of the tumor volume associated with the recovery of gonadal function to predict the necessity of long-term hormone replacement. From a statistical perspective, our study revealed that male patients with a larger pre-operative tumor volume have a greater risk of requiring post-operative long-term testosterone replacement (p = 0.0067, OR: 5.928, confidence interval [CI]: 1.637–21.465). Our study also revealed that the patients in the LTG (with a pre-operative tumor volume ≥7 cm^3^) exhibited an even greater risk for requiring post-operative long-term testosterone replacement than the patients in the STG, as determined by chi-square analysis (p = 0.0012, OR: 7.772, CI: 2.090–28.904) and multivariate logistic regression analysis (p = 0.0144, OR: 7.944, CI: 1.512–41.749). Therefore, a patient with a larger tumor, especially one ≥7 cm^3^, will have a greater risk of post-operative hypogonadism. Additionally, as mentioned above, a larger tumor volume would tend to cause more severe pre-operative hypogonadism. Based on our study, patients with more severe pre-operative hypogonadism, especially when the testosterone level is <1.5 ng/mL, will have a greater risk for the need for post-operative testosterone supplementation according to chi-square analysis (p < 0.0001, OR: 14.706, CI: 3.484–62.500) and multivariate logistic regression analysis (p = 0.0015, OR: 17.544, CI: 2.976–100.000). Therefore, the pre-operative testosterone level could also be used as a predictor of the need for testosterone replacement even though it may be only a secondary effect of the tumor volume.

#### Intraoperative Resection Status and Residual Tumor Ratio

**Figure 2:**
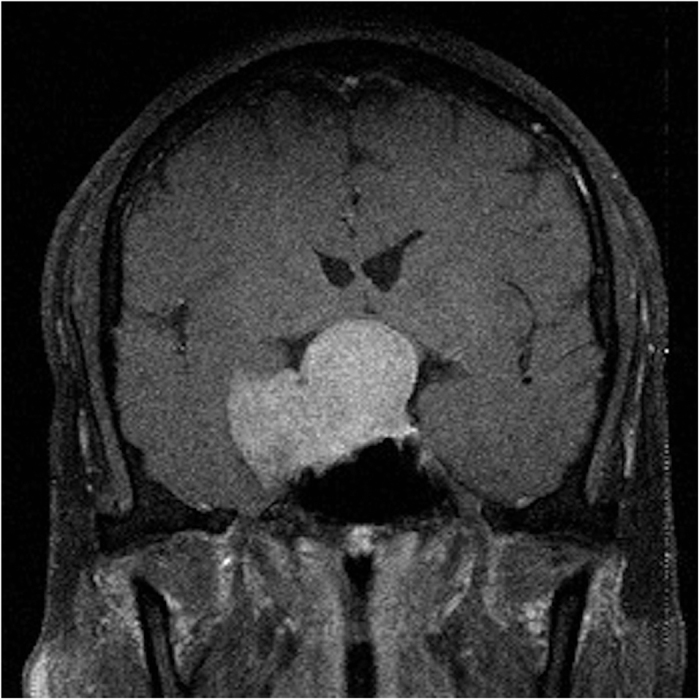
Patient 16 had a tumor that measured 33.51 cm^3^. It had invaded the paracavernous area; therefore, only partial removal could be achieved.

**Figure 3:**
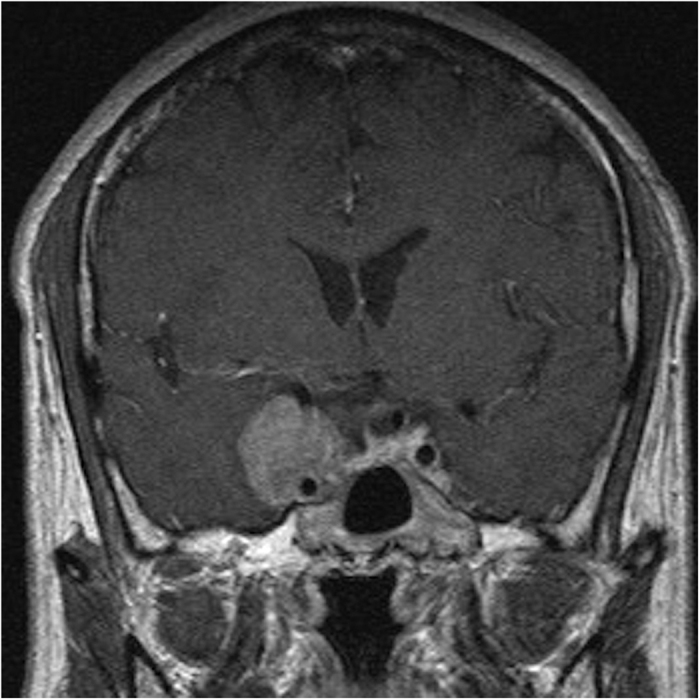
Most of the residual tumor resided in the paracavernous area, and the sella was extensively decompressed. However, the patient required long-term testosterone replacement possibly due to the large tumor volume and its effect on the pituitary gland.

We found that there was no significant difference between the GTR and STR groups in the need of post-operative long-term testosterone replacement by chi-square analysis (p = 0.2986) and multivariate logistic regression analysis (p = 0.1059). To explain the statistical results regarding GTR and STR, we assumed that we did our best to resect the tumor as much as possible during all operations to achieve optic nerve and pituitary gland decompression. In some cases, the operation was defined as STR because of the residual tumor tissue residing in the cavernous sinus rather than abutting the functional gland, and these patients did not require long-term testosterone supplementation. The reason for this outcome was that, in these STR cases, the pituitary gland was also greatly decompressed, as demonstrated by intraoperative computed tomography (CT) and post-operative MRI, which rendered long-term testosterone supplementation unnecessary, as described in the GTR cases. Therefore, we suggest that even in probable STR cases, the tumor should be resected as much as possible during the operation to reduce the residual tumor ratio and, most importantly, to achieve decompression of the sella. In contrast, some other patients who received STR did require long-term testosterone supplementation. We found that these patients had a much larger tumor volume and greater paracavernous invasion (Fig. 2), which indicates that they were at a higher risk for requiring long-term testosterone supplementation because of the much larger mass effect, as discussed in the previous section despite the decompression of the sella (Fig. 3).

Regarding the residual ratio, although it was found to be insignificant based on chi-square analysis (cut-off value: 0.22, p = 0.0515), univariate logistic analysis (cut-off value: 0.22, p = 0.0595), and multivariate logistic regression analysis (p = 0.1040), the stratification analysis, as shown in Table 10, demonstrated statistically significant differences regarding the higher residual tumor ratio in the need of post-operative long-term testosterone supplementation, which may suggest that more residual tumor ratio is a possible risk factor for requiring long-term post-operative testosterone replacement. From a statistical perspective based on aforementioned results, we noted that there was a statistically significant difference between more residual tumor ratio and the need of long-term testosterone replacement once the tumor volume was larger than the stratified tumor volume (e.g., ≥7 cm^3^) compared with a smaller tumor volume (<7 cm^3^). Similar results were found when the stratified volumes were at 8 and 9 cm^3^, although the difference was not so significant when the volume was ≥9 cm^3^ (p = 0.0429). These results indicate that, although the pre-operative tumor volume is the primary cause of post-operative hypogonadism, a more residual tumor ratio may also carry a greater risk of the requirement of long-term testosterone replacement. Based on this result, we should make every effort to resect the tumor as much as possible, especially when it reaches a significant volume (i.e., 7 cm^3^), to reduce the residual ratio to achieve pituitary gland decompression. This result is in accordance with our previous conclusion. Additionally, we found that there were no significant differences in the need of long-term testosterone replacement when using a stratified volume of 10 and 11 cm^3^. This situation may be attributed to the fact that when the tumor exceeds our optimal cut-off value of 7 cm^3^ too much or in the case of extremely large tumors with paracavernous invasion even after a GTR attempt, the patient still has a greater risk of post-operative hypogonadism despite adequate decompression of the sella because of the much larger pre-operative tumor volume and its irreversible impact on post-operative hypogonadism. Therefore, these statistical outcomes may suggest that not only the pre-operative tumor volume but also the residual ratio may carry higher risks for the need for post-operative long-term testosterone supplementation.

As mentioned above, in GTR and probable STR cases, the goal of surgery was to achieve decompression of the sella and to reduce the residual tumor ratio; however, excessively aggressive resection should be avoided because it not only causes damage to the pituitary gland because of the blind surgical manipulation but also does not help to restore post-operative long-term pituitary function. Additionally, according to our previous report^26^, the extent of resection could be assessed as reliably with intraoperative CT as with post-operative MRI. As a result, the degree of sella clearance, tumor resection ratio, and GTR/STR status could be determined via imaging as well as during the operation.

#### Second Surgery

Even though the multivariate logistic regression analysis showed a significant difference and a greater risk (OR: 183.160) in patients who underwent second operation (p = 0.0250) regarding the need of long-term post-operative testosterone replacement, the confidence interval ranged from 1.925 to 999.999, which may indicate that the result was not so convincingly significant. Two reasons for this result were hypothesized. First, the sample size may have been too small (only 7 cases). Second, we believe that the extreme significance of the pre-operative tumor volume and the testosterone level may have masked the importance of the second surgery. However, we still regard a second surgery as a potential risk factor for the need for long-term testosterone replacement not only because of the impact of recurrent/residual tumor itself but also because of the repeated surgical manipulations. However, further investigation should be performed by including more cases in the future.

In conclusion, regarding testosterone replacement in male patients, a larger pre-operative tumor volume (≥7 cm^3^), a lower pre-operative testosterone level (<1.5 ng/mL), and a higher residual tumor ratio are associated with a greater risk of the requirement of post-operative long-term testosterone replacement. Using intraoperative CT or MRI, after the sella is decompressed, we can terminate the surgical procedure, thus avoiding excessive, unnecessary, and inappropriate surgical manipulation of the pituitary gland. We can predict the need for post-operative testosterone replacement pre-operatively based on the tumor volume, testosterone level, and during the operation, we can eliminate unnecessary, harmful manipulation of the pituitary gland after the sella is decompressed.

### Study Limitations

We believe that a second surgery is a potential risk factor for the need for long-term testosterone replacement; however, the sample size was small, which is a limitation of our study. By including more cases in the future, we could obtain more significant statistical results regarding the risk of a second surgery or other potential risk factors.

## Methods and Materials

### Patient Population

Between 2004 and 2012, 45 male patients with NFPAs were enrolled in this prospective long-term study. All patients underwent endonasal endoscopic transsphenoidal surgery for tumor removal, and 7 patients required a second operation because of tumor recurrence. Pituitary function was assessed pre- and post-operatively. Routine post-operative MRI was performed within 3 months after surgery and annually thereafter. All experiments were performed in accordance with the relevant guidelines and regulations. The local ethics committee granted approval of the study, and informed consent was obtained in each case. Approval was also obtained from the Institutional Review Board of Chang Gung Memorial Hospital.

### Patient Subgroups

We divided the patients into different groups based on the pre-operative tumor volume, the pre-operative testosterone level, the resection status, and the residual ratio (V2/V1 = post-operative volume/pre-operative volume). By applying univariate logistic regression, we found that at volumes of 7 and 8 cm^3^, which yielded the best sensitivity and p value, and because we wanted to predict the need for long-term testosterone replacement, we had to choose the most sensitive value associated with the smallest tumor volume. Therefore, we could predict the need for long-term replacement at an earlier stage (smaller tumor volume). Conversely, when considering the pre-operative testosterone level, at levels of 1.3, 1.4, and 1.5 ng/mL at which the best sensitivity and p value existed, we had to choose the highest level to predict the need for long-term replacement at an earlier stage. Therefore, we determined a pre-operative tumor volume of 7 cm^3^ and a pre-operative testosterone level of 1.5 ng/mL as our cut-off values. Regarding the residual ratio, when the ratio was 0.22, although the p value did not reach a significant value, it approximately approached 0.05 (p value = 0.0595); therefore, we chose 0.22 as our cut-off value.

### Assessment of Pituitary Function

A complete pituitary function test based on clinical signs and symptoms was performed to evaluate pituitary function and to reveal pre- and post-operative pituitary deficiencies. Basal hormonal measurements were obtained for all patients, and the following criteria were used to define pituitary hormone deficiency^1^,^27^.

GH deficiency was defined as an IGF-I level below the reference range for age and sex^28^ and/or an insufficient increase in the GH level (absolute value <3 ng/mL) after stimulation during an insulin tolerance test. Hypocortisolism was diagnosed if the serum cortisol levels were low (<4.2 μg/dL) at 0800 h or below 1.7 μg/dL at 1600 h. Hypothyroidism was diagnosed if a subnormal serum-free T4 (FT4) level (<0.69 ng/dL) was associated with a low or normal TSH level (0.35–5.50 mIU/mL). In males, hypogonadism was diagnosed if the serum levels of testosterone were low (<2.4 ng/mL) in the presence of low or normal levels of gonadotropins (<10 IU/L). In postmenopausal women, hypogonadism was diagnosed if the serum LH and/or FSH levels were inappropriately low for the subject’s age (<1.5 mIU/mL). In premenopausal women, gonadotropin deficiency was diagnosed based on the presence of amenorrhea or oligomenorrhea and infertility, a low or low-normal basal level of gonadotropins (normal: LH, 2–16 mIU/mL; FSH, 2–10 mIU/mL) and persistently low estradiol levels (<30 pg/ml; <0.11 nmol/L). Diabetes insipidus was defined as polyuria not responding to fluid restriction but responding to vasopressin administration.

### Imaging Interpretation

The surgeon interpreted the pre- and post-operative MRI findings. Subsequently, the neuroradiologist, who was blinded to the prior interpretation, provided an independent retrospective evaluation of the MRI in an attempt to decrease the reporting bias. The post-operative MRI findings were interpreted as GTR if enhancement included an exclusively normal pituitary gland and granulation tissue or as STR if residual enhancements in areas other than the normal pituitary gland and granulation tissue were evident.

### Tumor Volume Calculation

To quantitatively define the tumor size and resection status, the volume of the tumor tissue was calculated based on the pre- and post-operative 1.5 T MRI using OsiriX software (www.osirix-viewer.com). We determined the pre-operative volume (V1), the post-operative volume (V2), and the residual tumor ratio (V2/V1), which was used to represent the completeness of the surgery. V1 and V2 were calculated as the averages of the tumor volume individually computed from the axial, sagittal, and coronal images. According to Arafah, 2.5 cm is a critical cut-off tumor diameter for the improvement of pituitary function, and the corresponding tumor volume is approximately (2.5)^3^/2 = 7.8125 cm^3^. Moreover, the univariate logistic regression showed that a volume of 7 cm^3^ was the best cut-off value, as mentioned above; thus, we used 7 cm^3^ as our cut-off value for the tumor volume. Tumors with a volume larger than 7 cm^3^ were categorized into the LTG; those measuring 7 cm^3^ or less were categorized into the STG. As previously noted, in cases defined as GTR, the volume obtained was associated with a normal pituitary gland and granulation tissue. To establish and standardize the average volume of the normal pituitary gland and the granulation tissue after GTR of a pituitary adenoma, we calculated the average volume of the residual pituitary gland and granulation tissue, which was regarded as the baseline value and ranged between 0.20 and 0.40 cm^3^ (mean ± 2SD). These data were obtained from 12 GTR patients in our previous report who were followed up for a long period after transsphenoidal pituitary surgery^26^.

### Endocrine and Radiological Follow-Up

The endocrine tests that were performed pre-operatively were repeated at 7 days; at 1, 3, 6, and 12 months after surgery; and annually thereafter. After resection of a pituitary tumor, packing materials, post-operative debris, thickened mucosa, and blood can interfere with imaging interpretation; however, these post-operative changes resolve within 3–4 months after surgery. Therefore, it is recommended to assess the effectiveness of surgery after approximately 3 months and annually thereafter for long-term follow-up after the initial surgery. The first post-operative MRI was performed within 3 months of surgery in most of our cases. Subsequent surveillance imaging studies were conducted at 1-year intervals for 2–3 years and then at longer intervals. However, patients with residual tumors received more frequent follow-ups.

### Hormone Replacement

The hormone replacement regimen depended on the laboratory data and the clinical symptoms. Because clinical symptoms are always subtle, we initiated hormone replacement therapy primarily based on the laboratory data. If an endocrine deficiency was found, adequate substitution was initiated using hydrocortisone, thyroxine, gonadal steroids, and desmopressin as appropriate. In our study, we focused on hypogonadism and prescribed testosterone supplementation for male patients with a serum testosterone level below 2.4 ng/mL or who exhibited clinical symptoms of hypogonadism. Hormone replacement for longer than 1 year was considered to indicate a “long-term” need for hormone supplementation.

### Statistical Methodology

The chi-square test and Fisher’s exact test for independence were used to determine the statistical significance of the differences in the need for post-operative long-term testosterone replacement between different categorical variables. Univariate logistic regression analysis was performed to analyze the continuous variables and to determine the best cut-off values of the pre-operative tumor volume, testosterone level, and residual tumor ratio. Multiple logistic regression analysis was performed to determine which variables and whether the chosen cut-off values independently predicted the need for long-term testosterone replacement. In all cases, a difference was considered significant if p < 0.05. SAS (Statistical Analysis System) software (version 9.3) was used (SAS Institute Inc., 100 SAS Campus Drive, Cary, NC 27513–2414, USA).

## Conclusions

The pre-operative tumor volume and pre-operative testosterone level significantly impacted post-operative hypogonadism and the need for hormone replacement, especially when the pre-operative tumor volume was ≥7 cm^3^ or testosterone level <1.5 ng/mL. The residual tumor ratio and degree of sella decompression may also influence the long-term need for testosterone supplementation. We advocate using volumetric MRI techniques and direct volume measurements to evaluate pituitary tumors, their impact on the pituitary gland, and the tumor resection ratio because these measurements can provide the most reliable results and can help surgeons to predict the degree of post-operative endocrine deficiency and the need for hormone replacement based on either the pre-operative tumor volume or to intraoperatively manipulate the degree of tumor resection.

## Additional Information

**How to cite this article**: Lee, C.-C. *et al.* Prediction of Long-term Post-operative Testosterone Replacement Requirement Based on the Pre-operative Tumor Volume and Testosterone Level in Pituitary Macroadenoma. *Sci. Rep.* **5**, 16194; doi: 10.1038/srep16194 (2015).

## Figures and Tables

**Table 1:** Subgroups of the 52 surgeries.

Characteristics	n
Age at surgery	
Mean (SD)	45.8 (14.6)
Range	12.2–73.3
Tumor volume (cm^3^)	
LTG	29/52 (55.8%)
STG	23/52 (44.2%)
Resection status	
STR	24/52 (45.8%)
GTR	28/52 (54.2%)
Residual ratio	
<0.22	40/52 (76.9%)
≥0.22	12/52 (23.1%)
Pre-operative testosterone level (ng/mL)	
<1.5	28/52 (53.8%)
≥1.5	24/52 (46.2%)
Mean pre-operative volume (cm^3^)	
LTG	15.67
STG	4.17
STR	13.71
GTR	8.21
Mean residual ratio	
LTG	0.17
STG	0.14
STR	0.26
GTR	0.07

LTG: large tumor group, STG: small tumor group, STR: subtotal tumor resection, GTR: gross total tumor resection.

**Table 2:** Hormone replacement in different groups.

	Long-term Replacement (−)	Long-term Replacement (+)
LTG (Large)	11/29 (37.9%)	18/29 (62.1%)
STG (Small)	19/23 (82.6%)	4/23 (17.4%)
STR (subtotal)	12/24 (50.0%)	12/24 (50.0%)
GTR (total)	18/28 (64.3%)	10/28 (35.7%)
Residual ratio <0.22	26/40 (65.0%)	14/40 (35.0%)
Residual ratio ≥0.22	4/12 (33.3%)	8/12 (66.7%)
Preoperative testosterone level <1.5 ng/mL	9/28 (32.1%)	19/28 (67.9%)
Preoperative testosterone level ≥1.5 ng/mL	21/24 (87.5%)	3/24 (12.5%)

LTG: large tumor group, STG: small tumor group, STR: subtotal tumor resection, GTR: gross total tumor resection.

**Table 3:** Univariate logistic regression: association and outcome prediction of *pre-operative tumor volume* → *Testosterone therapy* (1 year/needed vs. less than 1 year or none).

Tumor volume (cm^3^)	P values	Unadjusted odds ratio (95% CI)	Area under ROC curve	Sensitivity	1 – Specificity	Specificity
Cont.	0.0022	1.267 (1.089–1.474)	0.814	–	–	–
6	0.0104	6.333 (1.542–25.999)	0.682	0.8636	0.5000	0.5000
7	0.0022	7.772 (2.090–28.904)	0.726	0.8181	0.3666	0.6334
8	0.0022	7.772 (2.090–28.904)	0.726	0.8181	0.3666	0.6334
9	0.0066	5.333 (1.595–17.829)	0.697	0.7272	0.3333	0.6667
10	0.0019	7.041 (2.051–24.164)	0.724	0.6818	0.2333	0.7777
11	0.0006	13.000 (3.005–56.234)	0.745	0.5909	0.1000	0.9000
12	0.0009	16.794 (3.187–88.485)	0.739	0.5454	0.0666	0.9334
13	0.0018	13.997 (2.661–73.626)	0.717	0.5000	0.0666	0.9334

**Table 4:** Univariate logistic regression: association and outcome prediction of *residual tumor ratio* → *Testosterone therapy* (1 year/needed vs. less than 1 year or none).

Residual tumor ratio	P values	Unadjusted odds ratio (95% CI)	Area under ROC curve	Sensitivity	1 – Specificity	Specificity
Per 0.1	0.0996	1.398 (0.938–2.082)	0.536	–	–	–
0.06	0.5757	0.722 (0.231–2.257	0.538	0.4090	0.3333	0.6667
0.08	0.4251	0.637 (0.211–1.928)	0.556	0.5454	0.4333	0.5667
0.1	0.5750	0.729 (0.242–2.199)	0.539	0.5454	0.4666	0.5334
0.12	0.7459	0.833 (0.277–2.511)	0.523	0.5454	0.5000	0.5000
0.14	0.6942	1.250 (0.411–3.803)	0.527	0.5454	0.4000	0.6000
0.16	0.6942	1.250 (0.411–3.803)	0.527	0.5454	0.4000	0.6000
0.18	0.7561	1.196 (0.387–3.697)	0.521	0.4090	0.3666	0.6334
0.20	0.8205	1.143 (0.361–3.622)	0.515	0.3636	0.3333	0.6667
0.22	0.0595	3.714 (0.949–14.541)	0.615	0.3636	0.1333	0.8667
0.24	0.9640	N/A	0.636	0.2727	0.0000	1.0000
0.26	0.9640	N/A	0.636	0.2727	0.0000	1.0000

**Table 5:** Univariate logistic regression: association and outcome prediction of *pre-operative testosterone level* → *Testosterone therapy* (1 year/needed vs. less than 1 year or none).

Pre-operative testosterone level (ng/mL)	P values	Unadjusted odds ratio (95% CI)	Area under ROC curve	Sensitivity	1 – Specificity	Specificity
Cont.	0.0011	3.663 (1.675–8.000)	0.804	–	–	–
2.3	0.0107	16.129 (1.905–142.857)	0.694	0.9545	0.5666	0.4444
2.1	0.0074	18.519 (2.183–166.667)	0.711	0.9545	0.5333	0.4667
1.9	0.0053	10 (1.980–50.000)	0.705	0.9090	0.5000	0.5000
1.7	0.0061	7.246 (1.761–29.412)	0.698	0.8636	0.4666	0.5334
1.5	0.0003	14.706 (3.484–62.500)	0.782	0.8636	0.3000	0.7000
1.4	0.0003	14.706 (3.484–62.500)	0.782	0.8636	0.3000	0.7000
1.3	0.0003	14.706 (3.484–62.500)	0.782	0.8636	0.3000	0.7000
1.2	0.0008	8.772 (2.475–31.250)	0.747	0.7272	0.2333	0.7667
1.1	0.0009	8.547 (2.415–30.303)	0.741	0.6818	0.2000	0.8000
0.9	0.0122	4.808 (1.408–16.393)	0.673	0.5454	0.2000	0.8000
0.7	0.0063	6.494 (1.696–25.000)	0.683	0.5000	0.1333	0.8667

**Table 6:** Univariate analysis of categorical variables using chi-square and Fisher’s exact tests: association between factors and the need of long-tern testosterone therapy.

Characteristics	Testosterone therapy, n (%)		*Χ*^2^ tests, p value	Unadjusted odds ratio (95% CI)
	None or <1 year	≥1 year or needed		
Pre-operative tumor volume, 7 cm^3^			0.0012	7.772 (2.090–28.904)
<7 cm^3^ (STG)	19 (82.6)	4 (17.4)		
≥7 cm^3^ (LTG)	11 (37.9)	18 (62.1)		
Pre-operative testosterone level, 1.5 ng/mL			<0.0001	14.706 (3.484–62.5)
<1.5 ng/mL	9 (32.1)	19 (67.9)		
≥1.5 ng/mL	21 (87.5)	3 (12.5)		
Residual ratio, 0.22			0.0515	3.714 (0.949–14.541)
<0.22	26 (65.0)	14 (35.0)		
≥0.22	4 (33.3)	8 (66.7)		
Resection group			0.2986	0.556 (0.183–1.690)
STR	12 (50.0)	12 (50.0)		
GTR	18 (64.3)	10 (35.7)		
Second surgery			0.9999*	1.026 (0.205–5.133)
No	26 (57.8)	19 (42.2)		
Yes	4 (57.1)	3 (42.9)		

STR: subtotal tumor resection, GTR: gross total tumor resection, LTG: large tumor group, STG: small tumor group.

^*^Fisher’s exact test.

**Table 7:** Multivariate logistic regression: association between factors and the need of long-term testosterone therapy →*Testosterone therapy* (1 year/needed vs. less than 1 year or none) = *pre-operative tumor volume* + *pre-operative testosterone level* + *second surgery* (yes vs. no) + *resection status* (GTR vs. STR) + *residual tumor ratio*

Covariates	P value	Adjusted odds ratio (95% CI)
Tumor volume, per 5 cm^3^ increase	0.0067	5.928 (1.637–21.465)
Pre-operative testosterone level, per 0.5 ng/mL decrease	0.0101	3.155 (1.314–7.576)
Second surgery, yes vs. no	0.0250	183.160 (1.925–999.999)
Resection group, GTR vs. STR	0.1059	11.329 (0.598–214.777)
Residual tumor ratio, per 0.1 increase	0.1040	4.747 (0.726–31.051)

GTR: gross total tumor resection, STR: subtotal tumor resection.

**Table 8:** Multivariate logistic regression: association between factors and the need of long-term testosterone therapy →*Testosterone therapy* (1 year/needed vs. less than 1 year or none) = *pre-operative tumor volume* + *pre-operative testosterone level* + *second surgery* (yes vs. no)

Covariates	P value	Adjusted odds ratio (95% CI)
Tumor volume, per 5 cm^3^ increase	0.0082	3.771 (1.410–10.090)
Pre-operative testosterone level, per 0.5 ng/mL decrease	0.0055	2.703 (1.340–5.464)
Second surgery, yes vs. no	0.0257	119.014 (1.787–999.999)

**Table 9:** Multivariate logistic regression: association between factors and the risk/need of long-term testosterone therapy →*Testosterone therapy* (1 year/needed vs. less than 1 year or none) = *pre-operative tumor volume* (7 cm^3^) + *pre-operative testosterone level* (1.5 ng/mL) + *second surgery* (yes vs. no).

Covariates	P value	Adjusted odds ratio (95% CI)
Tumor volume, ≥7 vs. <7 cm^3^	0.0144	7.944 (1.512–41.749)
Pre-operative testosterone level,<1.5 vs. ≥1.5 ng/mL	0.0015	17.544 (2.976–100.000)
Second surgery, yes vs. no	0.1190	9.304 (0.563–153.640)

**Table 10:** Stratification analysis: association between factors [*residual tumor ratio*, *second surgery* (yes vs. no), *pre-operative testosterone level*] and risk of long-tern testosterone therapy, by multivariate logistic regression stratified by pre-operative tumor volume.

Stratification: pre-operative tumor volume	N	Area under curve	Residual tumor ratio, per 0.1 increase		Second surgery, yes vs. no		Pre-operative testosterone level, per 0.5 increase	
			P value	Adjusted OR(95% CI)	P value	Adjusted OR(95% CI)	P value	Adjusted OR(95% CI)
<7 cm^3^	23	0.855	0.2723	0.356 (0.056–2.251)	0.1923	37.097 (0.162–999.999)	0.1301	0.453 (0.163–1.263)
≥7 cm^3^	29	0.917	0.0367	4.713 (1.100–20.189)	0.9797	999.999 (0.001–999.999)	0.0255	0.248 (0.073–0.843)
<8 cm^3^	23	0.855	0.2723	0.356 (0.056–2.251)	0.1923	37.097 (0.162–999.999)	0.1301	0.453 (0.163–1.263)
≥8 cm^3^	29	0.917	0.0367	4.713 (1.100–20.189)	0.9797	999.999 (0.001–999.999)	0.0255	0.248 (0.073–0.843)
<9 cm^3^	26	0.854	0.3267	1.446 (0.692–3.019)	0.2245	20.260 (0.158–999.999)	0.0971	0.439 (0.166–1.161)
≥9 cm^3^	26	0.909	0.0429	4.336 (1.048–17.940)	0.9801	999.999 (0.001–999.999)	0.0236	0.237 (0.068–0.824)
<10 cm^3^	30	0.888	0.3335	1.436 (0.690–2.987)	0.1166	32.151 (0.421–999.999)	0.0438	0.401 (0.165–0.975)
≥10 cm^3^	22	0.862	0.0608	4.066 (0.983–17.620)	0.9980	3.387 (0.001–999.999)	0.0396	0.253 (0.068–0.937)
<11 cm^3^	36	0.914	0.3617	1.379 (0.691–2.751)	0.0850	75.607 (0.550–999.999)	0.0270	0.326 (0.121–0.880)
≥11 cm^3^	16	0.744	0.1855	3.104 (0.580–16.601)	N/A	N/A	0.1392	0.310 (0.066–1.464)

**Table 11:** Effect of transsphenoidal surgery in clinically nonfunctioning adenomas on pituitary function.

	No. of patients	Time after surgery for evaluation of pituitary function (months)	Suprasellar extension (%) Parasellar/infrasellar extension (%)	Pituitary: preoperative function	Pituitary: postperative function
				GH deficiency (%)	GH deficiency (%)
				LH/FSH deficiency (%)	LH/FSH deficiency (%)
				TSH deficiency (%)	TSH deficiency (%)
				ACTH deficiency (%)	ACTH deficiency (%)
				Hypopituitarism (%)	Hypopituitarism (%)
Arafah *et al.* (6)	26	0.2	80	100	85
			ND	96	65
				81	35
				62	38
				ND	ND
Dekkers *et al.* (12)	109	6	96	77	83
			36	75	90
				43	57
				53	60
				83	94
Nomikos *et al.* (8)	660	12	ND	ND	ND
			ND	77	65
				19	16
				35	18
				85	72
Alameda *et al.* (3)	51	ND	82	80	88
			48	62	57
				21	27
				19	19
				85	89
Wichers-Rother *et al.* (2)	109	1–6	ND	85	78
			ND	61	50
				31	34
				32	25
				ND	ND
Marazuela *et al.* (4)	35	2–6	80	88	82
			84	69	48
				23	20
				29	13
				69	ND
Comtois *et al.* (7)	126	ND	94	ND	ND
			33	75	70
				18	31
				36	29
				73	ND
Greenman *et al.* (31)	26	3–6	96	ND	ND
			42	78	46
				23	12
				43	50
				89	65

ND, Not documented.
